# Three-dimensional (3D) evaluation of liquid distribution in shake flask using an optical fluorescence technique

**DOI:** 10.1186/s13036-017-0070-7

**Published:** 2017-08-03

**Authors:** Amizon Azizan, Jochen Büchs

**Affiliations:** 1 Faculty of Chemical Engineering, Universiti Teknologi MARA, 40450 Shah Alam, Selangor Malaysia; 2 Aachener Verfahrenstechnik, Biochemical Engineering, RWTH Aachen University, Forckenbeckstrasse 51, 52074 Aachen, Germany

**Keywords:** Shake flask, Three dimensional (3D) liquid distribution, Leading edge of bulk liquid (LB), Tail of bulk liquid (TB), CFD, Circumferential liquid distribution, Maximum liquid height

## Abstract

**Background:**

Biotechnological development in shake flask necessitates vital engineering parameters e.g. volumetric power input, mixing time, gas liquid mass transfer coefficient, hydromechanical stress and effective shear rate. Determination and optimization of these parameters through experiments are labor-intensive and time-consuming. Computational Fluid Dynamics (CFD) provides the ability to predict and validate these parameters in bioprocess engineering. This work provides ample experimental data which are easily accessible for future validations to represent the hydrodynamics of the fluid flow in the shake flask.

**Results:**

A non-invasive measuring technique using an optical fluorescence method was developed for shake flasks containing a fluorescent solution with a waterlike viscosity at varying filling volume (V_L_ = 15 to 40 mL) and shaking frequency (*n* = 150 to 450 rpm) at a constant shaking diameter (d_o_ = 25 mm). The method detected the leading edge (LB) and tail of the rotating bulk liquid (TB) relative to the direction of the centrifugal acceleration at varying circumferential heights from the base of the shake flask. The determined LB and TB points were translated into three-dimensional (3D) circumferential liquid distribution plots. The maximum liquid height (H_max_) of the bulk liquid increased with increasing filling volume and shaking frequency of the shaking flask, as expected. The toroidal shapes of LB and TB are clearly asymmetrical and the measured TB differed by the elongation of the liquid particularly towards the torus part of the shake flask.

**Conclusion:**

The 3D liquid distribution data collected at varying filling volume and shaking frequency, comprising of LB and TB values relative to the direction of the centrifugal acceleration are essential for validating future numerical solutions using CFD to predict vital engineering parameters in shake flask.

**Electronic supplementary material:**

The online version of this article (doi:10.1186/s13036-017-0070-7) contains supplementary material, which is available to authorized users.

## Background

Shake flasks and stirred bioreactors are amongst the popular choice in the development of biotechnological processes. Shake flasks are widely used for biological screenings and process optimizations for the simplicity in geometry, orderly parallel experiments, ease of operation and its low operating cost [1]. In the last years, the main engineering parameters, for instance volumetric power input [2–6], mixing time [7], gas-liquid mass transfer coefficient [8–12], hydromechanical stress [2, 8, 13] and effective shear rate [14] have been characterized. These vital engineering parameters are significantly governed by the hydrodynamic state in the system in which they influence the yield and quality of the final product in a bioprocess [15].

Computational Fluid Dynamics (CFD) modeling has been used to predict some of the above mentioned engineering parameters like volumetric power input and gas-liquid mass transfer coefficient. Zhang et al. [16] and Li et al. [17] simulated two-phase flow fields locating the free interfacial surface area in shake flasks. They predicted the volumetric mass transfer and compared it to the work of Maier and Büchs [9]. It deepens insights about the fluid dynamics within a shake flask. The volume-of-fluid (VOF) method has been applied to solve the continuity equation of Navier Stokes [16, 17] for free surface flows which can also be found in other orbitally shaken (microplate or cylindrical well) and wave-mixed bioreactor systems [18]. However, in some cases, the models derived may not completely elucidate the real situation in the bioreactors. This might be due to the assumptions of the limited boundary conditions, the applied turbulence models chosen and the spatial and temporal discretization of the transport phenomena to a finite-space grid used [15, 18]. Therefore, to answer the numerical uncertainties, CFD models have to be validated or calibrated by measured data.

Many investigations using CFD have been done with stirred bioreactors [15, 18–20] and orbitally shaken bioreactors [16, 17, 21–25]. However, to get measured data to validate the CFD simulation proves to be a big challenge. In a stirred bioreactor, the interior fluid flow of the bulk liquid is induced by impeller stirring and shows a quite complex pattern. The typical gassed liquid-air dispersions increase the complexity level of fluid flow determination [26]. The details may comprise of the flow of the impeller region, either simple shear, diverging, converging or rotational vortex flows [27]. Therefore, detailed fluid velocity data from the interior of the bulk liquid in the stirred vessel are needed. For that, a precise and reliable technique is required. The fluid flow data for the bulk liquid can be measured by using contactless and contacting measurement techniques which are e.g. particle image velocimetry [26, 28, 29], laser doppler anemometry [30] and hot film anemometer [31]. In short, the complete 3D characterization of fluid flows in stirred bioreactors requires measurement techniques that are difficult, highly sophisticated and very time-consuming [19].

There were also some attempts to characterize the hydrodynamics of the flow behavior in shake flasks by using particle image velocimetry and laser light tracer methods with different geometries (unbaffled, baffled and coiled) at shaking frequencies of 150 rpm [32], 200 rpm [32] and 80 to 100 rpm [33]. Mixing dynamics in relation to velocity fields and turbulent intensities were reported.

Shake flasks are different from stirred bioreactors. The former develop a specific shape of the rotating bulk liquid inside the bioreactors, resulting from the rotating centrifugal field. Thus, this shape of the liquid provides an excellent source of information for the validation of CFD simulations. In relation to gas-liquid mass transfer, the hydrodynamics of fluids with waterlike viscosity in shake flask can be described by a ‘two-sub-reactor model’ [10–12], defining mass transfer through the liquid film on the flask wall and base (falling film system) and the bulk liquid rotating within the flask (mixed stirred tank reactor system). Based on this ‘two-sub-reactor model’ the mass transfer area (a) and the mass transfer coefficient (k_L_) were modeled and compared with measured values at a broad range of operating conditions. The agreement was found to be quite satisfactory. In stirred bioreactor, the liquid position with a horizontal surface is well defined, nonetheless, containing no information. However, as mentioned above, for shake flasks, it is assumed that the shape and the position of the rotating bulk liquid relative to the centrifugal acceleration contain enough information to validate CFD calculations. Therefore, a more complicated measuring approach may not particularly be required.

Previously, the liquid distribution in shake flasks has theoretically been estimated by a simplifying approach disregarding viscosity of the liquid and assuming an ideal frictionless fluid motion [9, 10, 34]. It was reported that the intersection between the rotational paraboloid of the liquid and the inner wall of the shake flasks produces a symmetrical body for the liquid. Validation was undertaken by comparing the computed maximum liquid height to the measured values from photographs [9]. It was also shown that the predicted absolute values of the gas-liquid mass transfer area agree well with those measured by the accelerated sulfite system, if the liquid film formed on the inner glass wall of the flask by the rotating bulk liquid was also taken into account [9]. However, a qualitative comparison of photographs with simulation results of the simplified model of liquid distribution [34], clearly elucidate differences. Whereas the simulated liquid distributions are inherently symmetrical, the real distributions are clearly asymmetrical, showing an extended elongated tail. The same approach was also used in large disposable orbitally shaken cylindrical vessels formulating a well-defined liquid distribution of the bulk liquid, resulting from an intersection between the rotational liquid paraboloid and the cylindrical bioreactor wall [35].

It is the aim of this paper to extend and improve the assessment of the rotating bulk liquid distribution in shake flask. The measuring principle is derived from the previous work reported by Ottow et al. [22] by using a non-invasive optical fluorescence technique. The mentioned work [22] reported successful measurement of the well defined leading edge and tail of the rotating bulk liquid at one single height (14.74 mm). In this current work, angular circumferential rotating bulk liquid positions at 14 specific heights are detected, collecting extensive measured data of liquid distribution in shake flasks. Such data can provide useful input for future CFD modeling representing the fluid dynamics in shake flasks. Consequently, the CFD predictions of volumetric power input, mixing time, gas-liquid mass transfer coefficient, hydromechanical stress and effective shear rate in shake flask can be verified. This paper shows three-dimensional (3D) presentations of the liquid distribution by the angular positions of the leading edge and the tail of the bulk liquid. Extensive data obtained at a specific shaking diameter, different shaking frequencies and filling volumes were based on the circumferential rotating bulk liquid at the wall around the shake flask. It should be noted that in this study, the information of the depth of the rotating bulk liquid perpendicular to the flask wall was not assessed. Doing that would be much more difficult as a usual shake flask is optically not a perfectly symmetrical body and calibration of the liquid depth would be extremely demanding.

## Method

### Experimental setup of the optical fluorescence measurement technique

A 5 μM fluorescein solution (Fluorescein Acid Yellow 73, Aldrich, Germany) was used as the fluorescent model solution, buffered with a 100 mM phosphate buffer (Na_2_HPO_4_.2H_2_O ≥ 99%; NaH_2_PO_4_.H_2_O ≥ 98%; Roth, Germany) and adjusted to pH 8 with 5 M sodium hydroxide (NaOH).

**Fig. 1:**
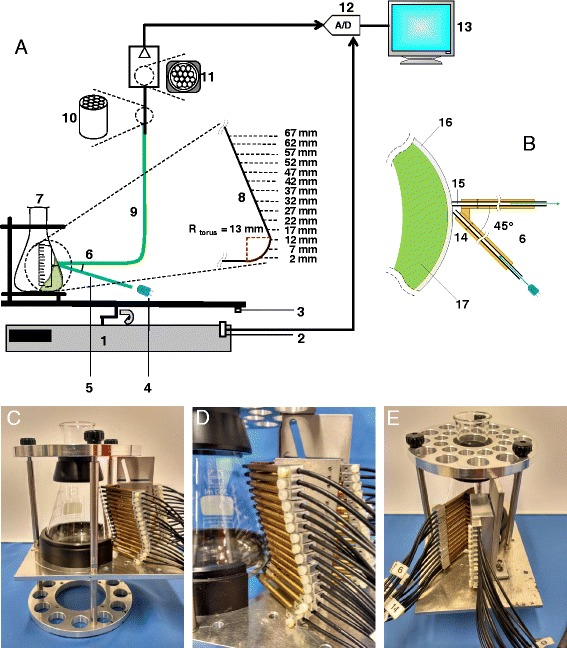
Liquid distribution measurement in shake flasks using an optical fluorescence technique. **a** shows a schematic diagram of the liquid distribution measurement setup for 250-mL shake flask. **b** shows a top view of the optical fiber arrangement at the side of the shake flask wall. **c**, **d** and **e** show a side view, close-up view and 45^o^ distant view of the optical fiber arrangement to the shake flask, respectively. 1) Orbital shaker. 2) Hall sensor. 3) Magnet for triggering. 4) Light-emitting diode (LED) supplying excitation blue light (465 nm). 5) Optical fiber channeling the excitation blue light to the shake flask. 6) 45^o^ angle distance of the optical fiber arrangement (seen from top view). 7) 250-mL shake flask clamped onto the orbital shaker’s table. 8) Enlarged view of the 14 different measurement positions to the side of the shake flask wall. Fourteen pairs of optical fibers were arranged vertically at these specified different heights from the base of the shake flask. 9) Optical fiber channeling the emission light (515 nm) from the shake flask to the photomultiplier tube (PMT). 10) Enlarged view of the bundle of 14 optical fibers. 11) Enlarged view of the PMT’s light sensitive area attached with a bundle of 14 optical fibers. 12) Data acquisition module (analog-to-digital converter). 13) Computer. 14) Brass holder for optical fibers. 15) Transparent core of optical fiber. 16) Shake flask wall. 17) Rotating fluorescent aqueous solution

Figure 1a and b show schematic diagrams of the measuring setup for the liquid distribution. Fourteen pairs of plastic optical fibers (8: Poly (methyl methacrylate) (PMMA) with cladding material, Conrad Components, Germany) were held in brass holders (14). Two corresponding holders were separated by an angle of 45^o^ (6). The pairs were arranged vertically to the side of a 250-mL shake flask wall (7: Schott Duran, Germany). The height started at 2 mm to 67 mm (with an increment of 5 mm) from the base of the shake flask. Each pair has a 2-mm optical fiber (15) with a core of a 1-mm transparent fiber, one (5) from the light source (light emitting diode) to the shake flask having the fluorescein solution (17) and the other one (9) was from the shake flask to the light receiver (photomultiplier tube) (11).

Blue Indium Gallium Nitride (InGaN) light-emitting diodes (4: Kingbright, Germany) supplied blue excitation light at a peak wavelength of 465 nm. They were alternately switched on at different heights. All of the 14 pairs of optical fibers channeling the emission lights from the shake flask were bundled (10) to be in contact to a 15-mm width (11) of the light sensitive area at a photomultiplier tube (Hamamatsu H7827–001, Japan). The blue excitation lights illuminated the fluorescein solution which resulted in emission lights at the peak wavelength of 518 nm. A long pass optical filter (OG 515, Reichmann, Germany) that was positioned prior to the photomultiplier tube, filtered the signals received to the wavelength of above 515 nm only. Subsequently, a low noise amplifier and an analog-to-digital converter (A/D) (12) amplified and converted the analog signals, using a data acquisition card. The digitized signals were expressed as output voltage (V) and recorded by a computer (13) for further interpretations.

A 250-mL shake flask is comprised of the torus with a radius of 13 mm and the conical upper part. The shapes of the torus and conical parts of the shake flask were calculated accordingly as proposed by [34]. The 250-mL shake flask was pretreated with a boiling 20% nitric acid (HNO_3_) solution to create hydrophilic conditions at the inner flask wall. The washed and cooled pretreated flask with the attached optical fiber arrangement was then clamped onto the orbital shaker’s table (1: Kuhner, Switzerland) having a hall sensor (2). During a rotation, as the hall sensor was in alignment with a magnet (3) fixed at the shaker’s table, the recording of the signals (output voltage) started. For better viewing of the optical fiber arrangement on the shake flask, Fig. 1c–e show a side view, a close-up photo and a 45^o^ distance view of the measurement setup, respectively.

Using the above described setup, experiments were conducted with varying filling volumes (V_L_ = 15 to 40 mL with an increment of 5 mL) of fluorescein solution at varying shaking frequencies (*n* = 150 to 450 rpm with an increment of 25 rpm). The shaking diameter was kept constant at 25 mm.

### Liquid distribution detection

**Fig. 2:**
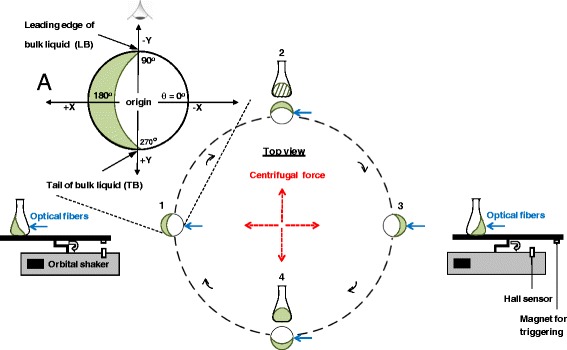
Shifts of the positions of the rotating bulk liquid in a 250-mL shake flask (40-mm radius) for one complete orbital rotation (illustration is not-to-scale). The start point of a clockwise rotation is at the position 1 when the magnet triggers the hall sensor located at the shaker. Centrifugal forces (red arrows) induced by the orbital rotation exert the bulk liquid towards the wall side of the shake flask along the orbital diameter. The leading edge of a bulk liquid (LB) defines the circulating shift around the shake flask’s circumference having 360^o^ circular angle. Tail of a bulk liquid (TB) follows LB circulation in the shake flask. The eye symbol indicates the reference viewing position for the three-dimensional plot as shown in Figs. 4b and 5. **a** depicts the close-up illustration of the shake flask orientation on angular circular position of liquid detection (θ) [^o^] and Cartesian coordinates of X and Y axes (+X and –X, +Y and –Y). The positive and negative X and Y orientations will be useful for 3D liquid distribution plots in the subsequent figures

The measurement setup on the shaker’s table rotated in a clockwise direction (top view). As illustrated in Fig. 2, the uniform rotational motion created centrifugal forces outward along the circumference of the shaking diameter orbit on the bulk liquid (not-to-scale drawn dashed red lines). For better interpretation, 90^o^ incremental angular positions were set as a basis to the liquid’s location in a shake flask. The location of the set of optical fibers was referred to be at the 0^o^ plane. For a better understanding of the liquid distribution to be later translated into three-dimensional (3D) plots (see Fig. 4), an eye viewpoint was chosen. The eye viewpoint was positioned to be at the 90^o^ plane as shown in the figure.

The liquid distribution measuring setup recorded the signals every time the liquid moved to the front and passed by the optical fiber’s location. Ten complete rotational motions starting from position 1 were recorded to give an average data of liquid distribution in shake flask. The front of the rotating bulk liquid was depicted as the leading edge of the bulk liquid (LB). On the other hand, the end of the bulk liquid was defined as the tail of the bulk liquid (TB). The recorded liquid distribution was a plot of output voltage [V] against the 360^o^ angle of the liquid’s location in the shake flask [^o^].

**Fig. 3:**
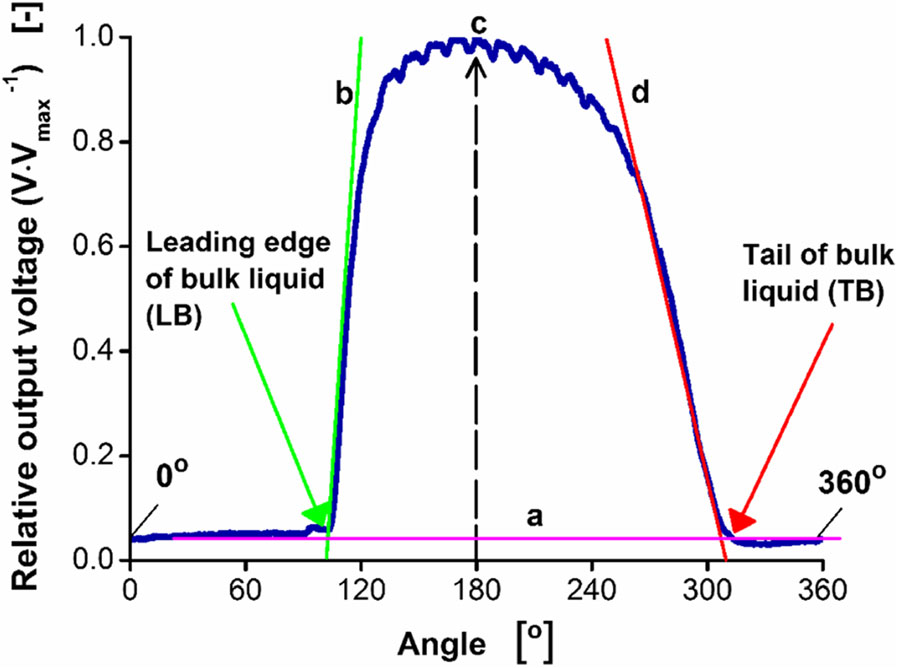
Example of measured liquid distribution data (360^o^ circular angle) plotted as output voltage [V] relative to the maximum values of the output voltage [V_max_] for a 250-mL shake flask rotating at 200 rpm shaking frequency and 25 mm shaking diameter. The recorded liquid distribution data shown is the results of the averaged values from 10 rotations observed at the position 1 from Fig. 2. The flask was filled with 25 mL of a 5 μM fluorescein solution with 100 mM phosphate buffer (pH 8). The measurement was taken at a height of 22 mm from the base of the shake flask. The intersecting points between the lines of ‘a’ and ‘b’ as well as between the lines of ‘a’ and ‘d’ lead to the leading edge (LB) and the tail of the bulk liquid (TB), respectively. LB and TB determined are identified at 104.84^o^ and 306.35^o^, respectively

Figure 3 shows an example of a liquid distribution data plot for V_L_ = 25 mL of 5 μM fluorescein solution in a 250-mL shake flask, rotated at *n* = 200 rpm and d_o_ = 25 mm. It was measured at a height of 22 mm from the base of the shake flask. In this figure, the liquid distribution plot was divided into the following sequence: (starting from the left) the low horizontal line (a: minimal slope and minimal output voltage), abrupt ascending curve (b: high positive slope with increasing output voltage), indicating the leading edge (LB) of the bulk liquid, high horizontal curved line (c: minimal slope and maximal output voltage) and the descending curve (d: high to moderate negative slope with decreasing output voltage) indicating the tail of the bulk liquid (TB). LB and TB angular positions were determined by determining the points of the intersection between (a) and (b) as well as between (a) and (d), respectively. This was done by applying linear regression method on the LB and TB regions. The method was justified by a goodness of fit (R^2^) which was higher than 95%. For instance, in Fig. 3, the determined LB and TB angular positions were identified at 104.84^o^ and 306.85^o^, respectively.

## Result and Discussion

### Three-dimensional plot

**Fig. 4:**
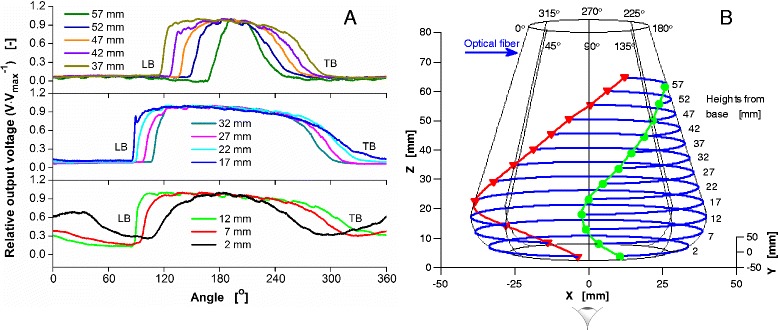
Measured liquid distribution data (360^o^ circular angle) for a 250-mL shake flask containing 40 mL of an aqueous 5 μM fluorescein solution with 100 mM phosphate buffer (pH 8). Measurement conditions are 400 rpm of shaking frequency and 25 mm of shaking diameter. **a** shows the output voltage [V] relative to the maximum values of the output voltage [V_max_] detected. The leading edge (LB) and tail of the bulk liquid (TB) are observed at varying heights starting from 2 mm to 57 mm (with an increment of 5 mm) from the base of the shake flask. The heights of 2 mm to 12 mm refer to the torus of the flask; the heights of 17 mm to 57 mm refer to the conical part (see Fig. 1). **b** depicts the three-dimensional (3D) bulk liquid positions for LB (green solid circle and line) and TB (red solid triangle and line) at the shake flask’s wall based on determined angle values [^o^] at varying heights. The location of the optical fiber is referred at the 0^o^ circular angle of the shake flask. The direction of the centrifugal force is pointing from left to right. The LB and TB are observed at azimuth and elevation angles of 180^o^ and 6^o^, respectively

Having all the data collected about the liquid distribution at specific operating conditions of the flask, the 3D liquid distribution plots of the bulk liquid on the wall of the shake flask were prepared. Figure 4a shows an example of the liquid distribution data at the heights of 2 mm to 57 mm from the base of the shake flask with an increment of 5 mm for V_L_ = 40 mL, *n* = 400 rpm and d_o_ = 25 mm. More examples of liquid distribution data are also presented (Additional file 1: Figure S1). The figure depicts the relative output voltage (V/V_max_) value which was defined as the ratio of the measured output voltage to the maximum output voltage detected at each height, versus the 360^o^ circular angle of the shake flask’s circumference. The torus and conical part were referred to as V/V_max_ readings at the heights of 2 mm to 12 mm and the heights of above 17 mm, respectively. As observed, the width of the rotating bulk liquid circulating the shake flask became smaller to the higher measurement position at the conical part starting from the height of 17 to 57 mm. From the liquid distribution plots (Fig. 4a), LB and TB angular positions were determined according to the method as illustrated in Fig. 3. These angular positions were translated into the corresponding 3D liquid distribution in the shake flask (Fig. 4b). The three-dimensional shape of the shake flask was formed based on 8 vertical planes of 45^o^ angle each. The location of the optical fibers was at the 0^o^ position. The 3D plot orientation is in accordance to the eye position viewpoint as shown in Fig. 2. The chosen eye position allows clearer view of the leading edge (LB) and the tail (TB) of the bulk liquid for the chosen parameters selected. As illustrated in Fig. 4b, the LB was plotted as green solid circle and TB as red solid triangle at each height. The green and red lines connecting the LB and TB angular positions, respectively, represented the circumferential liquid distribution on the shake flask’s wall. The curve fitting lines were obtained by using a shape preserving interpolation model, specifically the Piecewise Cubic Hermite Interpolating Polynomial (PCHIP) as available in MATLAB.

Figure 4b observes that the exerted rotating bulk liquid concentrated towards the 180^o^ plane in the direction of the centrifugal force. On the other hand, the tail of the bulk liquid was as if dragging behind the leading edge of the bulk liquid. According to the earlier simplifying liquid distribution model [34], the shape of the rotating bulk liquid should be symmetrical for both the LB and TB. However, the measured liquid distribution data clearly did not reveal a symmetrical LB and TB relative position. Instead, the TB angular positions were elongated to an additional 90^o^ covering a longer circumferential area. A preliminary comparison of measured data generated in this work and corresponding CFD simulation, originating from the work of Ottow et al. [22] shows a satisfactory agreement (Additional file 2 Figure S2). This implies that the depth of the rotating bulk liquid on the flask wall is obviously in reality lower than predicted by the simplifying non-viscosity model [34], what is not an unexpected finding.

### Effect of filling volume to maximum liquid height (H_max_), positions of leading edge (LB) and tail of rotating bulk liquid (TB)

**Fig. 5:**
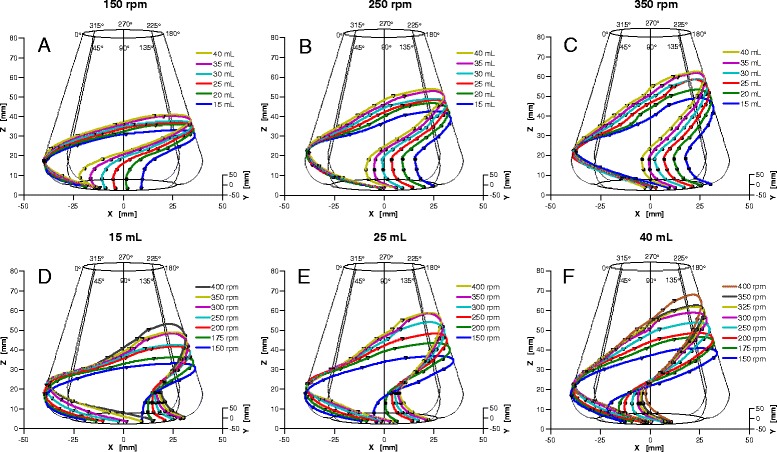
Three-dimensional (3D) illustrations of the bulk liquid position in a 250-mL shake flask containing 5 μM fluorescein solution with 100 mM phosphate buffer (pH 8), rotated at 25 mm of shaking diameter, with the same viewing direction as in Figs. 2 and 4. The leading edge (LB) and tail of the bulk liquid (TB) are observed at azimuth and elevation angles of 180^o^ and 6^o^, respectively. **a, b** and **c** indicate 3D illustrations of the bulk liquid with varying filling volume and shaking frequency of V_L_ = 15 to 40 mL (at an increment of 5 mL) and *n* = 150, 250 and 350 rpm, respectively. **d, e** and **f** show 3D illustrations of the bulk liquid with varying shaking frequency and filling volume of *n* = 150 rpm to 400 rpm (at an increment of 25 and 50 rpm) and V_L_ = 15, 25 and 40 mL, respectively

In this investigation, the filling volume was varied while all the other parameters were held constant. Figure 5a–c depict the compilations of the 3D liquid distribution plots for varying filling volumes (V_L_ = 15 to 40 mL with an increment of 5 mL), at three different shaking frequencies (*n* = 150, 250 and 350 rpm) and at a constant shaking diameter (d_o_ = 25 mm).

Figure 5a shows the change of the maximum liquid height (H_max_) (in the z direction) as well as the LB and TB positions with varying V_L_ at *n* = 150 rpm. At V_L_ of 15 to 40 mL (with an increment of 5 mL), H_max_ values are between 25.9 to 33.8 mm from the base of shake flask, respectively. It is obvious that H_max_ increases as V_L_ increases. It is also observed that the LB positions sequentially shift with increasing V_L_ into the clockwise direction (left), relative to the centrifugal acceleration. On the other hand, despite the increasing V_L_, TB positions, particularly at the lower torus part, do not differ as much as that seen at the conical upper part of the shake flask.

The above findings are confirmed by Fig. 5b and c with n of 250 and 350 rpm, respectively. The H_max_ for V_L_ of 15 to 40 mL (with an increment of 5 mL) is recorded to be from 35.4 mm to 47.0 mm and from 42.0 mm to 56.1 mm, for *n* of 250 and 350 rpm, respectively. Visibly, the rise of H_max_ is influenced relatively to the increasing V_L_. Additionally, higher V_L_ also shifts the LB and TB positions except for the TB positions at the torus part of the shake flask, as described before. The LB and TB shifts are fairly consistent relative to the increase of V_L_.

### Effect of shaking frequency to maximum liquid height (H_max_), positions of leading edge (LB) and tail of rotating bulk liquid (TB)

Figure 5d–f show 3D liquid distribution plots of the rotating bulk liquid at a fixed d_o_ of 25 mm, with varying *n* = 150 to 400 rpm and at specific V_L_ of 15, 25 and 40 mL, respectively. Fig. 5d reports on the change of the H_max_ for a V_L_ of 15 mL at varying *n*. H_max_ values are between 25.9 and 46.1 mm are recorded for *n* of 150 to 400 rpm, respectively. It is concluded that H_max_ increases as *n* increases. The LB positions shift sequentially following the counter clockwise direction (right), relative to the centrifugal acceleration, as *n* increases. The phenomena illustrates as such that for a constant V_L_, the bulk liquid is pushed with higher *n* upward and inward, to the conical and torus parts of the shake flask, respectively. In contrast to what is seen for TB positions at constant *n* and varying V_L_ (see Fig. 5a–c), the TB positions distancing themselves a little apart from each other, particularly at the torus part of the shake flask.

Figure 5e and f with V_L_ of 25 and 40 mL, respectively, confirmed the above assessments. It is clearly seen that as *n* increases, H_max_ also increases. As in Fig. 5e, the H_max_ values are between 29.8 mm to 52.0 mm for *n* of 150 to 400 rpm (with an increment of 50 rpm), respectively. Whilst in Fig. 5f, for *n* of 150 to 400 rpm, the H_max_ values are from 33.8 mm to 61.4 mm, respectively.

## Conclusion

An extended non-invasive measurement method for assessing the liquid distribution in shake flasks by using an optical fluorescence technique was developed. It consists of 14 sets of optical fibers, 14 light-emitting diodes and one photomultiplier tube, employed for all 14 measurement positions and a fluorescein solution. The 3D circumferential distributions of the rotating bulk liquid were analyzed at varying circumferential heights starting from the base of the shake flask (torus) and extended upward to the conical part. A preliminary comparison of measured data generated in this work and corresponding CFD simulation shows a satisfactory agreement. Leading edge and tail of bulk liquid closely resemble circumferentially in shake flask to the CFD simulation. The information contained in the systematically collected data is very valuable for calibrating and validating Computational Fluid Dynamics (CFD) simulations, to better understand the hydrodynamic flow of fluid in shake flasks. This approach is essential for the numerical determination of some vital engineering parameters for example volumetric power input, mixing time, gas-liquid mass transfer coefficients, hydromechanical stress and effective shear rate in bioprocess development using shaking flasks. In a succeeding paper, varying liquid distributions CFD calculations utilizing the measured data from this work, initiated at higher viscosities will be reported.

## Additional files

Additional file 1: Figure S1. Other examples of the measured liquid distribution data (360^o^ circular angle) for a 250-mL shake flask containing 25 mL of a 5 μM fluorescent solution with 100 mM phosphate buffer (pH 8) at 25 mm shaking diameter. **A, B, C** and **D** indicate the liquid distribution measurement conditions of 15 mL and 300 rpm, 20 mL and 200 rpm, 25 mL and 150 rpm, and 30 mL and 350 rpm, respectively. The figures show the output voltage [V] relative to the maximum values of the output voltage [V_max_] detected. The leading edge (LB) and tail of the bulk liquid (TB) are observed at varying heights starting from 2 mm to 47 mm (with an increment of 5 mm) from the base of the shake flask. The heights of 2 mm to 12 mm refer to the torus of the shake flask while the heights of 17 mm to 47 mm refer to the conical part (as seen in Fig. 4). (PDF 109 kb)

Additional file 2: Figure S2. Computational Fluid Dynamics simulation [22] and three-dimensional (3D) liquid distribution plot from the introduced optical fluorescence technique. **A** shows CFD results generated by FLUENT simulations for 25 mL water at 300 rpm shaking frequency, 25 mm shaking diameter in a 250-mL shake flask. Viscosity, density and contact angle used are 0.001003 kg/ms, 998.2 kg/m^3^ and 20^o^, respectively. **B** depicts the measured 3D liquid distribution in 250-mL shake flask containing an aqueous 5 μM fluorescein solution with 100 mM phosphate buffer (pH 8). Data points were collected at varying heights from the base of the shake flask from 2 mm to 42 mm. The black circle and triangle symbols indicate the leading edge of the bulk liquid (LB) and tail of the bulk liquid (TB) for the liquid moving in a clockwise direction. The azimuth and elevation of both **A** and **B** are at 57^o^ and 9.6^o^, respectively. (PDF 84 kb)

## Abbreviations

3D: Three-dimensional
CFD: Computational Fluid Dynamics
d_o_: Shaking diameter
H_max_: Maximum liquid height
LB: Leading edge of bulk liquid
M: Molar
mL: Milliliter
mm: Millimeter
mM: Millimolar
*n*: Shaking frequency
Na_2_HPO_4_.2H_2_O: Disodium hydrogen phosphate dihydrate
NaH_2_PO_4_.H_2_O: Sodium dihydrogen phosphate monohydrate
nm: Nanometer
rpm: Revolution per minute
TB: Tail of bulk liquid
µM: Micromolar
V: Output voltage
V_L_: Filling volume
V_max_: Maximum output voltage
