# Supplementary figures and images for: Three-dimensional (3D) evaluation of liquid distribution in shake flask using an optical fluorescence technique
Source: J Biol Eng. 2017 Aug 3;11:28. doi: 10.1186/s13036-017-0070-7 (PMC5541408; doi:10.1186/s13036-017-0070-7)

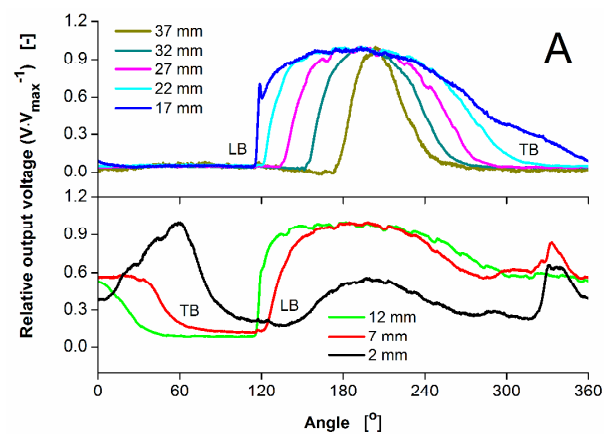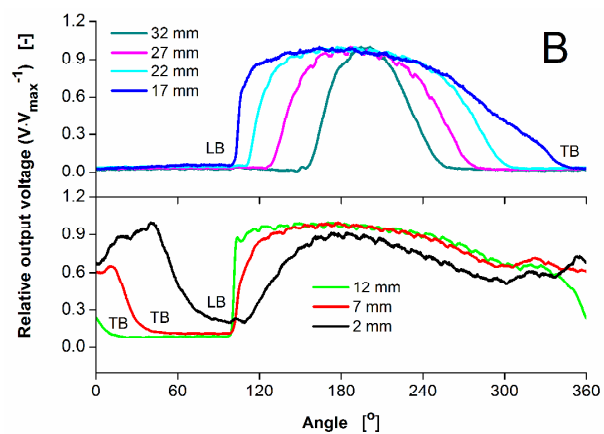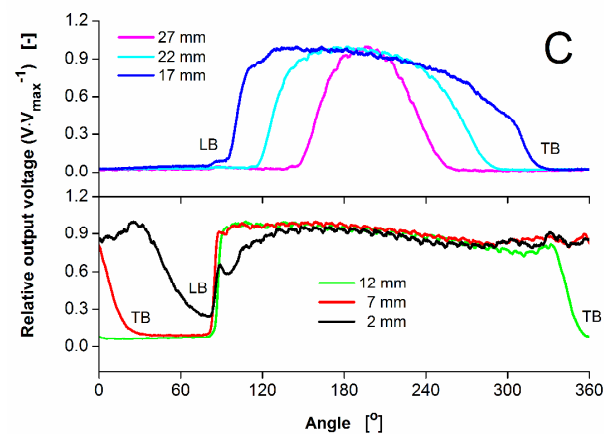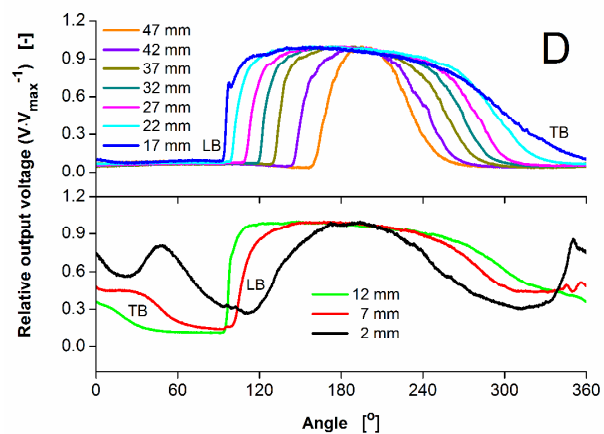

Supplement: Supplementary file 1 — Other examples of the measured liquid distribution data (360o circular angle) for a 250-mL shake flask containing 25 mL of a 5 μM fluorescent solution with 100 mM phosphate buffer (pH 8) at 25 mm shaking diameter. A, B, C and D indicate the liquid distribution measurement conditions of 15 mL and 300 rpm, 20 mL and 200 rpm, 25 mL and 150 rpm, and 30 mL and 350 rpm, respectively. The figures show the output voltage [V] relative to the maximum values of the output voltage [Vmax] detected. The leading edge (LB) and tail of the bulk liquid (TB) are observed at varying heights starting from 2 mm to 47 mm (with an increment of 5 mm) from the base of the shake flask. The heights of 2 mm to 12 mm refer to the torus of the shake flask while the heights of 17 mm to 47 mm refer to the conical part (as seen in Fig. 4). (PDF 109 kb) [file 13036_2017_70_MOESM1_ESM.pdf]

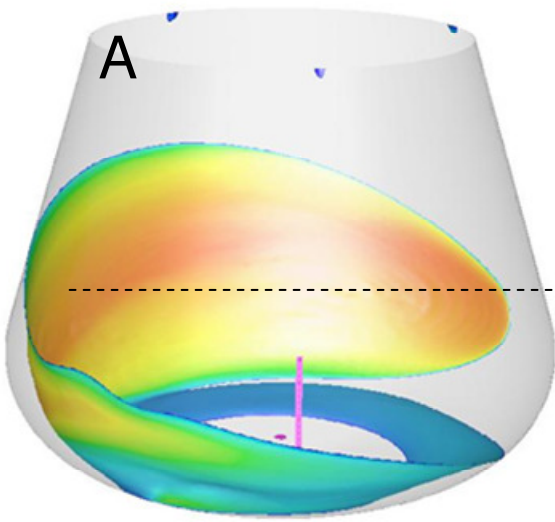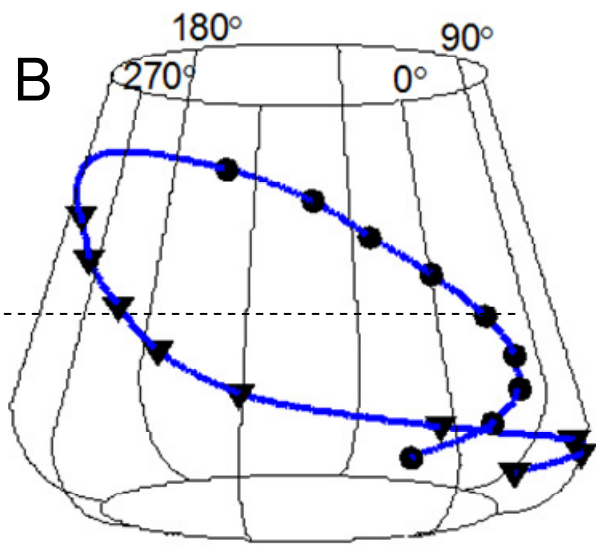

Supplement: Supplementary file 2 — Computational Fluid Dynamics simulation [22] and three-dimensional (3D) liquid distribution plot from the introduced optical fluorescence technique. A shows CFD results generated by FLUENT simulations for 25 mL water at 300 rpm shaking frequency, 25 mm shaking diameter in a 250-mL shake flask. Viscosity, density and contact angle used are 0.001003 kg/ms, 998.2 kg/m3 and 20o, respectively. B depicts the measured 3D liquid distribution in 250-mL shake flask containing an aqueous 5 μM fluorescein solution with 100 mM phosphate buffer (pH 8). Data points were collected at varying heights from the base of the shake flask from 2 mm to 42 mm. The black circle and triangle symbols indicate the leading edge of the bulk liquid (LB) and tail of the bulk liquid (TB) for the liquid moving in a clockwise direction. The azimuth and elevation of both A and B are at 57o and 9.6o, respectively. (PDF 84 kb) [file 13036_2017_70_MOESM2_ESM.pdf]
